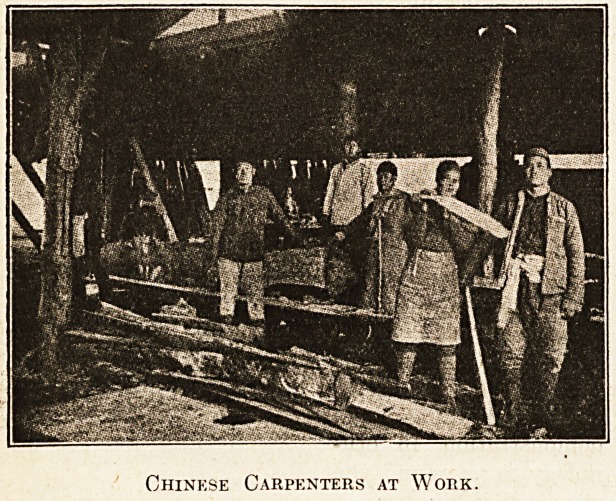# Many Races at the War

**Published:** 1918-11-02

**Authors:** 


### MANY RACES AT THE WAR.

### II.—With Native Labour Corps in France.

By A MEDICAL OFFICER.

On arrival at our destination we found almost every native race under the sun represented in the busy scene before us. In addition to our Fijians and the Chinese there were "Cape-boys" and other South Africans, fez-capped Egyptian "fellahs," West Indians of all colours, and Pathans, Ranchis, Santals, and other tribes from India; making as strikingly variegated a picture as the camouflaged tents they lived in.

Camouflaging was very evident everywhere, and the monster guns and armoured cars and even railway trucks were covered with vivid streaks and patches of brown, white, and green. We very quickly found the advantages of this where our own comfort was concerned, as the Hun night-birds were soon over us, searching about in the moonlight for suitable spots to drop their "eggs." This became in time such a common occurrence that even the natives ceased to pay much attention to them, beyond getting into dug-outs whenever possible. But in the beginning some curious incidents occurred. On one occasion a number of Chinese were drawn up at midday on parade when an enemy 'plane suddenly appeared and started dropping bombs. They were quickly given the order to dismiss, but until they received the order they stood firm as to their feet, but wildly shaking their fists up at the intruder and hurling strange Chinese oaths at it. On another occasion, when a duel was going on in mid-air, a small group of them did not scatter with the rest to dug-outs as ordered, but had to be almost dragged in. It appears that their national spirit of gambling had been roused and they were waiting to see the result of their bets on this wonderful foreign "kite-flying."

The Fijians stood the bombing as well as anybody, but during one of the earlier attacks there happened to be five of them up at the isolation hospital with chicken-pox, four being in one bell tent, and the fifth by himself in another tent some little distance off. He waited patiently while two enormous bombs fell, one to the right of him and one to the left, but when a third dropped about fifty yards behind him his discretion overcame his valour, and with one wild yell he dashed across the field like a hare, and sprang straight into bed with one of his comrades, to the consternation of them all.

The "Gyppies" also made valiant attempts to dig themselves in, but their dug-outs were hardly scientific, being only about eighteen inches in depth. It was no uncommon sight to see a pair of hind legs and a blanket, well exposed to any missile, the owner—like the ostrich—feeling quite secure now that his head was buried in the sand. It was not long, however, before good sand-bagged dug-outs were constructed for all troops, and any dangers except from a direct hit were reduced to a minimum.

All the same we had some very bad raids while I was in that part of France, and I shall always remember three successive nights when camps of Chinese, British, and finally even Germans (prisoners) were in succession badly knocked about. We had a very busy time at our big hospital that week, three operating-tables going practically night and day, amputating, patching up, and generally endeavouring to repair the damages done. One of our first cases of gas gangrene came in with the Chinese lot, a man who had been treated at the small camp hospital for apparently only a simple shell wound of the thigh. It was not long before the leg began to look swollen and the man's general condition to look bad; and on further opening it up we found the typical crepitations and dark-coloured flesh that afterwards became such a well-known feature of this sort of case. Early and large doses of sodium bicarbonate were found to be very beneficial in counteracting the acidosis in these gas gangrenes, but only too often amputation was necessary to save life.

Fijians in Fatigue Dress.

It was really quite a demonstration in ethnology to go round the wards of "Number — General." In addition to about a thousand beds for British troops there were separate marquees for patients from all the native races that were at work in the very large area that "fed" this hospital. In the Fijian ward could be seen the ever-cheerful "fuzzy-head" lying back between the unaccustomed sheets; or else a little group of two or three dressed in hospital "blues" sitting on a bench playing a native game at cards, or trying their best to keep up a conversation with one of their Australian friends who had dropped in from another ward. Australians and New Zealanders they always regarded as "next-door neighbours from home," Sydney being only a small matter of some 2,000 miles from their own coral lands!

After a little while many of them were able to speak very fair English, but when they first arrived there were only about three men who knew more than half-a-dozen words. One of these, however, was a well-educated young chief from a big New

The previous article appeared on October 26, p. 69.

**Many Races at the War**—(*continued*).

Zealand college, while another was the son of one of the leading men of the Colony, a native Member of the Legislative Council. This young man had been at Oxford when war broke out, and being keen on "doing his bit" had joined the famous "Foreign Legion," fighting side by side with some of the bravest sons of France for eighteen months in the trenches. Wounded twice and invalided home to Fiji he had insisted on coming out again when this contingent of his own people had been raised. Eventually he had to be again repatriated after a very bad bout of pneumonia, a complaint to which these South Sea Islanders were unfortunately too prone.

As long as they were well wrapped up and moving freely about they were all right, but in their happy-go-lucky island fashion they would throw off their great-coats and unbutton their tunics directly they got heated, with bad results to their chests. Curiously enough, the actual cold they did not mind at all, and were delighted to join in a snowball fight, after they had got over their first astonishment at the strange stuff!

Chinese Carpenters at Work.

All sorts of the "Tommies'" pastimes they were keen on (even to playing the inevitable "House"); and they had one great day when some big aquatic sports were organised. Accustomed to swim almost before they could walk they would have swept the board of all the prizes, but unfortunately it was a late autumn day with a biting cold wind sweeping across the lagoon, with the result that though they pluckily entered for everything going they were generally left behind breathless and blue with cold, to their great chagrin.

The Hospital Staff as Censors.

The Egyptians, like the Fijians, were very keen on sports, but kept much more to themselves, and although they could often be seen indulging in a game of football they inclined more to their own pastimes, a native variant of the old English bouts with quarter-staffs being one of the most popular. This game caused them many bruises on the shins, and one would sometimes see three or four of them at the same time go hopping and limping to the native orderly at the Egyptian section of the hospital for some "bruise medicine." This section was very nicely laid out with marquees surrounded by bright flower-beds, neat little sign-boards everywhere, and a polished French '75 shell-case as a gong outside the dining-tent. There was a special Arabic-speaking M.O. with a knowledge of the country to look after the Egyptians, just as the other native races had each their own medical officer, so that the duty of censoring the patient's home letters (which was always done by the hospital staff) was not such an amazingly intricate thing as it might have been. I remember one day on which letters written in Chinese, Fijian, Arabic, Hindustani, and Welsh appeared among the heap to be censored!

A Competition and its Result.

We had some curious accident cases while I was at this hospital. On one occasion three or four Chinamen and one Fijian were brought in as a result of a little difference between them. It appeared that the Fijians had to unload bales of hay from a ship and the Chinese simultaneously to stow them into a goods train waiting alongside. The spirit of both races was aroused and a keen competition set in. Gradually the mountain of bales that the Fijians had unloaded increased in size and gradually also increased the wrath of the Chinese, who could not get them fast enough into the train. The ultimate result was that four Chinese got black eyes and cut heads, and the Fijian had the lens of one eye dislocated with a half-brick! This Fijian was a huge fellow, with an ever-cheerful smile on his face and a Royal Humane Society riband on his breast (for very gallantly saving life at night in the shark-infested waters of Suva Harbour). He threatened to tear the offending Chinaman into little bits and eat him after the manner of his forefathers, but his anger soon cooled, and luckily his eyesight was not permanently damaged.

Why there were not more accidents among the Chinese heaven only knows, for they used to do the most absurd things. For instance, a Chinaman would be quietly standing up fishing on the canal bank when all of a sudden, apparently without rhyme or reason, he would topple over headfirst into the water, only to scramble out again with a smile and an "Allee litee." And one morning after a big raid a cheerful Celestial was seen crossing the parade ground joyfully swinging an unexploded aerial torpedo that he had picked up! There was a scramble in all directions until he had safely deposited it outside the sergeant's tent.

They were curious creatures and never quite understood the ways of the white foreign devils. The question of inoculation against enteric greatly perturbed them, as they had a fixed idea that it

(*Continued on page* 93.)

### Many Races at the War (*concluded from page* 90).

was some subtle endeavour to render them sterile; and their M.O.—who had lived for twenty-five years in China—had the greatest difficulty in dispelling this illusion. By the way, the definite entry of China into the war on the side of the Allies was a great day for this M.O., as hitherto his chest had been bare of adornment; but after he had opened his *Times* that day he disappeared into his tent, to come forth again a rainbow blaze of colours and decorations, from the "Order of the Bountiful Harvest" downwards, which he was now entitled to display.

Life was very good and very amusing in those days, and never without its interest and excitement; but the depths of winter were soon upon us, and it was thought that the South Sea Islanders, at all events, would be better in a warmer spot; so regretfully we had to make the long track across France to a southern port, and our adventures there I must reserve for some other day.

## Figures and Tables

**Figure f1:**
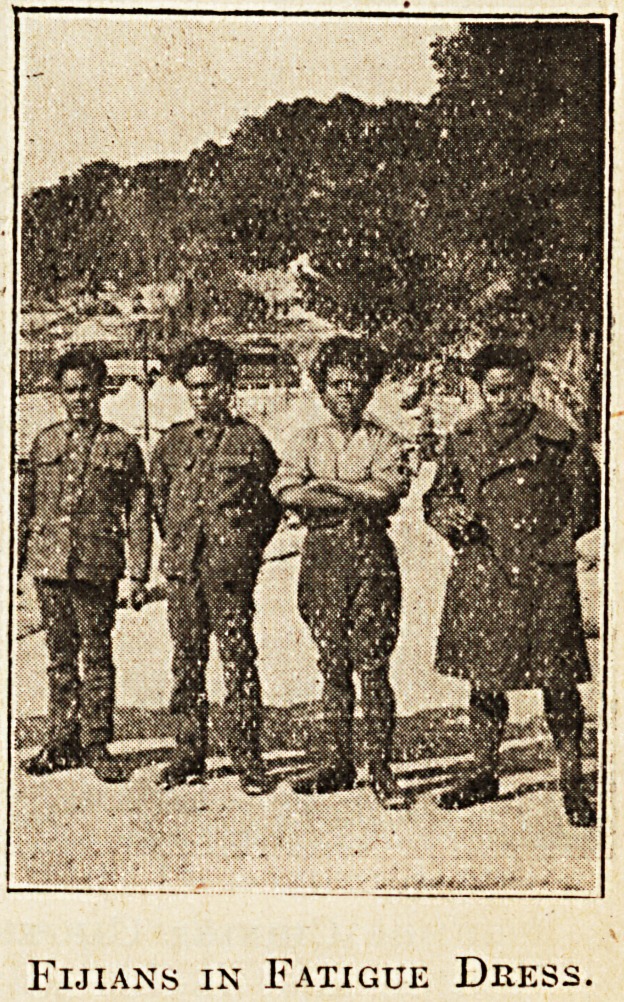


**Figure f2:**